# Practice Patterns of Treatment Strategy of Limited-Stage Small-Cell Lung Cancer: Survey of Chinese Oncologists

**DOI:** 10.3389/fonc.2022.872324

**Published:** 2022-05-12

**Authors:** Chang Xu, Meng Li, Xuwei Cai, Shuanghu Yuan, Jianzhong Cao, Shuchai Zhu, Ming Chen, Nan Bi, Xiao Hu, Jiancheng Li, Wei Zhou, Ping Wang, Lujun Zhao, Ningbo Liu

**Affiliations:** ^1^Department of Radiation Oncology, Tianjin Medical University Cancer Institute and Hospital, National Clinical Research Center for Cancer, Key Laboratory of Cancer Prevention and Therapy, Tianjin’s Clinical Research Center for Cancer, Tianjin, China; ^2^Department of Radiation Oncology, Konggang Branch of Tianjin Cancer Hospital, Tianjin, China; ^3^Department of Radiation Oncology, Fudan University Shanghai Cancer Center, Shanghai, China; ^4^Department of Radiation Oncology, Shandong Cancer Hospital and Institute, Shandong First Medical University and Shandong Academy of Medical Sciences, Jinan, China; ^5^Department of Radiation Oncology, Shanxi Cancer Hospital and the Affiliated Cancer Hospital of Shanxi Medical University, Taiyuan, China; ^6^Department of Radiation Oncology, The Fourth Hospital of Hebei Medical University, Shijiazhuang, China; ^7^Department of Radiation Oncology, Sun Yat-sen University Cancer Center, Guangzhou, China; ^8^Radiology Department, National Cancer Center/National Clinical Research Center for Cancer/Cancer Hospital, Chinese Academy of Medical Sciences and Peking Union Medical College, Beijing, China; ^9^Department of Radiation Oncology, Institute of Cancer Research and Basic Medical Sciences and University Cancer Hospital, Chinese Academy of Sciences, Zhejiang Cancer Hospital, Zhejiang Provincial Key Laboratory of Radiation Oncology, Hangzhou, China; ^10^Department of Radiation Oncology, Fujian Provincial Cancer Hospital, Clinical College of Fujian Medical University, Fuzhou, China; ^11^Department of Radiation Oncology, Chongqing University Cancer Hospital, Chongqing, China

**Keywords:** LS-SCLC, thoracic radiotherapy, hyper-fractionation, dosing, lung cancer

## Abstract

**Background:**

Thoracic radiotherapy (TRT) with concurrent chemotherapy is the standard treatment of limited-stage small-cell lung cancer (LS-SCLC). However, there is still a controversy surrounding the treatment strategy especially optimal dosing and fractionation schedule. Current practice patterns among Chinese oncologists are unknown.

**Materials and Methods:**

We surveyed 212 Chinese oncologists using a questionnaire including 50 questions designed by experienced oncologists. Questions covered demographic data, treatment recommendations, and self-assessed knowledge of guidelines or key clinical trials for SCLC. The chi-square test and Fisher’s exact test were utilized to describe the result of the study.

**Results:**

The response rate was 97% (207/212). Of all the respondents, 69% preferred TRT QD, 29% preferred BID, and 2% chose HFRT. For those who prefer TRT QD, 72% preferred a total dose of 60 Gy, followed by 15% opting for 66 Gy, 12% for <60 Gy, and 1% for 70 Gy. Of those who prefer BID, 79% preferred a total dose of 45 Gy, with 4% choosing 30 Gy, 8% choosing 50 Gy, 7% choosing 54 Gy, and 2% choosing >54 Gy. Regarding PCI, 82% of participants believed that PCI should be performed when treatment is completed and 13% believed that PCI should begin immediately after concurrent chemoradiotherapy. As for other therapies, 26% of participants choose concurrent anti-angiogenic therapy during SCLC treatment, and 49% recommended small-molecule TKI as the main anti-angiogenic therapy.

**Conclusion:**

Substantial variation exists in how Chinese oncologists approach TRT dosing and fractionation for LS-SCLC. Almost 70% of respondents reported administering TRT QD more often in daily work. The most common doses were 60 Gy QD and 45 Gy BID.

## Introduction

Small-cell lung cancer (SCLC) is a highly malignant neuroendocrine tumor with strong invasiveness and rapid progression and accounts for about 15% of all lung cancers, and the incidence of limited-stage small-cell lung cancer (LS-SCLC) is about 33,000 people per year (1, 2). Thoracic radiation (TRT), which has been demonstrated to improve overall survival (OS) in LS-SCLC, is the standard treatment for LS-SCLC when combined with chemotherapy (3), while substantial variation was found in the dosing and fractionation for TRT in practice.

In 1999, a survey from ECOG (INT 0096) showed that while twice-daily TRT of 1.5 Gy in 30 treatments improved survival compared with conventional TRT, it still had a higher rate of local failure, as well as hematological and pulmonary side effects (4). However, in 2017, the CONVERT trial confirmed no differences in survival or major toxicity between the 66-Gy QD regimen and the 45-Gy BID regimen (5). Based on the results of INT 0096, NCCN guidelines have recommended one of two treatment strategies: BID TRT in 1.5-Gy fractions over 3 weeks for a total dose of 45 Gy or once-daily (QD) TRT in 2.0-Gy fractions over 6 to 7 weeks for a total dose of 60–70 Gy (6). Given the ongoing debate, a survey of 309 American radiation oncologists was designed to analyze the substantial variation in the practice patterns of LS-SCLC treatment in 2018. The study claimed that most radiation oncologists preferred QD over BID fractionation and more than three quarters administered QD TRT more often to their patients. Influenced by NCCN guidelines, the overwhelming majority of those who preferred BID TRT recommended a total dose of 45 Gy, while for QD TRT the preferred dose varied from 60 to 70 Gy (7).

More recent assessments have been presented in response to the controversy. A multicenter retrospective review reported in the 2020 ASTRO annual meeting showed that a total of 804 patients with LS-SCLC was followed from 2000 to 2016 and divided randomly into 3 groups by different treatment strategies, including hypofractionated RT using 40 Gy in 15 fractions with once-daily treatment, standard RT using 50 Gy in 25 fractions with once-daily treatment, and hyperfractionated RT using 45 Gy in 30 fractions with twice-daily treatment. The analysis revealed no differences in OS across three commonly used RT regimens, while the use of hyperfractionated RT appeared to have a tendency of survival advantage (8). Bjorn Henning Gronberg et al. investigated 176 patients and claimed that compared with BID TRT with a total dose of 45 Gy, high-dose BID TRT of 60 Gy in 40 fractions showed a substantial benefit in 2-year survival (70.2% vs. 46.1%) and a significantly longer median overall survival (41.6 vs. 22.9 months) (9). Another phase III trial [CALGB 30610 (Alliance)/RTOG 0538.] reported in the 2021 ASCO annual meeting demonstrated that compared with 45-Gy BID TRT, high-dose QD TRT to 70 Gy did not significantly improve OS. Most grade 3+ hematologic and non-hematologic adverse events were similar between cohorts (10). Moreover, there was also a meta-analysis aimed at analyzing the treatment suitability of stereotactic ablative radiotherapy (SABR) in LS-SCLC which showed that for inoperable early-stage, node-negative SCLC, SABR is locally effective (nodal and distant failure rates were 17.8% and 26.9%, respectively) with limited toxicity (grade 1, grade 2, and grade 3 toxicity rates: 12.6%, 6.7%, and 1.4%, respectively. No grade 4 or 5 events were observed) (11).

Given the ongoing debates and progress of studies, we designed an online survey including the time and fractionation schedule of TRT for LS-SCLC, choices of prophylactic cranial irradiation (PCI), and angiogenesis inhibitors to learn how Chinese oncologists treat patients with LS-SCLC.

## Materials and Methods

### Survey Instrument Development

Relying on the Chinese Anti-Cancer Association, we surveyed 212 Chinese oncologists using an online questionnaire including 50 questions designed by experienced oncologists. The questions covered demographic data, treatment recommendations including the timing of TRT, dose fractionation schedule, PCI, and angiogenesis inhibitor use. The questions also covered self-assessed knowledge of key clinical trials or guidelines of LS-SCLC, including NCCN guidelines, INT 0096study, CONVERT trial, and RTOG 0538 trial. The duration since residency training related to SCLC treatment is also included in the self-evaluation. All the participants were asked to provide their answers for each question and choose the reasons prepared. An “other” option was also provided for the participants to type their reasons.

### Data Collection

The data were collected through an online questionnaire developed by Wenjuanxing software (https://www.wjx.cn). The questionnaire link was sent to oncologists across the country to participate in this project using email. Invitations were sent in August 2021 and closed in October 2021.

### Statistical Analysis

Data analysis was conducted using the SPSS 25.0 software (IBM Corp. Released 2017. IBM SPSS Statistics for Windows, Version 25.0. Armonk, NY: IBM Corp.). The chi-square test was used to identify differences between groups for categorical variables. Fisher’s exact test was utilized to compare the correlation between two categorical variables. *P*-value <0.05 indicated that the results were statistically significant.

## Results

### Survey Respondents

We sent 3,495 email addresses and received 548 undeliverable/failed automatic replies, 207 completed responses, and 5 inapplicable ones. Among the 207 participants, 206 are clinical oncologists from various levels of hospitals in China, and one is a student majoring in radiation oncology. Considering the rigor and credibility of the research, only the responses from 206 oncologists were analyzed. Among the 206 participants, there are 174 radiation oncologists, 31 are engaged in medical oncology, and one is an oncological surgeon. Of all the participants who completed the questionnaire, 5.8% of oncologists were from grade II hospitals while 94.2% were from grade III hospitals of which 86% oncologists were from grade III level A hospitals. The classification of all hospitals is based on the Chinese 3-tier classification system that recognizes a hospital’s ability to provide medical care and medical education and conduct medical research. Of all the 206 oncologists, 76% were practicing for over 10 years after completing residency training, while 13% practiced for 5–10 years and 11% for 5 years. The characteristics of 206 oncologists who completed the survey are summarized in **Table 1**.

**Table 1:** Demographic information for oncologists who completed the survey (n = 206).

Variable	Respondents, n (%)
**Classification of institutions**	
** Tertiary hospital**	26 (12.6)
** Grade II hospital**	12 (5.8)
** Grade III level A hospital**	168 (81.6)
**Professional title**	
** Chief physician**	79 (38.2)
** Associate chief physicians**	73 (35.3)
** Attending physicians**	39 (18.8)
** resident**	15 (7.7)
**Practice region**	
** North**	33 (15.9)
** Central**	24 (11.6)
** East**	85 (41.1)
** South**	7 (3.4)
** Northeast**	34 (16.9)
** Northwest**	11 (5.3)
** Southwest**	12 (5.8)
**Duration since residency training (years)**	
** <5**	19 (9.7)
** 5-10**	28 (13.5)
** >10**	159 (76.8)
**Number of SCLC patients treated per year in the department**	
** 10-40**	72 (34.8)
** 41-70**	62 (30.4)
** 71-100**	42 (20.3)
** >100**	30 (14.5)
**Number of SCLC patients treated per year by the oncologists own**	
** 10-40**	158 (76.7)
** 41-70**	33 (16.0)
** 71-100**	8 (3.9)
** >100**	7 (3.4)

### Timing of TRT With Chemotherapy Cycles

In our questionnaire, 100% of participants recommended TRT in LS-SCLC patients. More than half of participants (65.5%) preferred to begin TRT after two cycles of chemotherapy, 26.7% preferred one cycle, and 7.8% preferred to start 3 cycles or later. When asked at what time most of their patients with LS-SCLC started TRT in actual practice, 66.5% of participants answered 2 cycles, 26.2% answered 1 cycle, and 7.3% said in 3 cycles or later. There was a statistical significance between the preferred start time of TRT and some of the demographic characteristics of participants, including practice setting (Fisher’s exact *P* = 0.002), the number of SCLC patients treated in the department (Fisher’s exact *P* = 0.001), and the number of SCLC patients treated by their own (Fisher’s exact *P* = 0.007). There were no statistically significant correlations between the preferred start time of TRT and other demographic characteristics of respondents, including geographic location (Fisher’s exact *P* = 0.9) and years since residency training (Fisher’s exact *P* = 0.5). However, the preferred start time was not strongly correlated with the actual start time of the TRT (Kendall tau rank correlation *P* = 0.08). For nearly a half of respondents (43%), the actual start time of TRT differed from their preferred time, in which 47% of oncologists started TRT later than they preferred to, while 53% started earlier. Oncologists in high-level hospitals are more likely to start TRT after two cycles of chemotherapy and have more stable choices in actual practice.

When asked whether they preferred to choose concurrent chemoradiotherapy or sequential chemoradiotherapy, nearly 90% of participants recommended concurrent chemoradiotherapy, and only 10% preferred sequential chemoradiotherapy. Those who recommended concurrent chemoradiotherapy suggested that concurrent chemoradiotherapy can improve overall survival compared with sequential chemoradiotherapy. However, in practice, 25% of 206 oncologists cannot complete concurrent chemoradiotherapy when treating patients with LS-SCLC, indicating that there are actual difficulties that exist in concurrent chemoradiotherapy in practice.

### Fractionation Schedule for LS-SCLC

**Figure 1:**
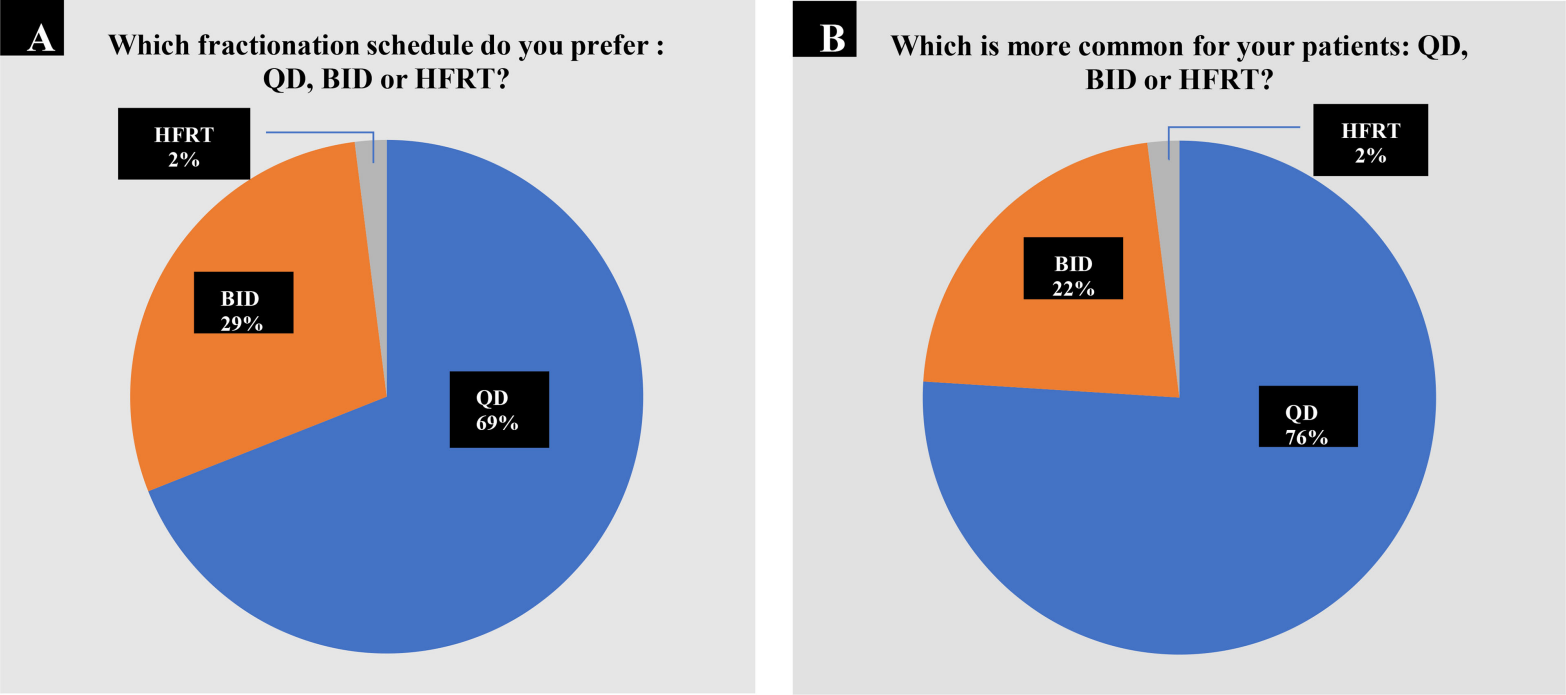
Choices of fractionation schedule of TRT. **(A)** 143 out of 206 oncologists (69.4%) preferred TRT once daily (QD); 60 participants (29.1%) preferred twice daily (BID) compared with 3 who preferred HFRT. **(B)** In actual practice, 157 out of 206 respondents (76.2%) chose QD TRT; 44 (21.3%) chose BID TRT combined with 5 oncologists (2.4%) who chose HFRT.

Our questionnaire provided three options for the radiotherapy fractionation schedule: QD, BID, and HFRT. Approximately 69% of all the respondents preferred TRT QD compared with 29% who preferred BID fractionation and only 2% who chose HFRT (**Figure 1A**). When asked which TRT schedule was more commonly used when they treat patients with LS-SCLC in actual practice, 76% chose QD, 22% said BID, while only 2% selected HFRT (**Figure 1B**). There were no statistically significant correlations between the preferred fractionation schedule and demographic characteristics of respondents, including practice setting (Fisher’s exact *P* = 0.24), geographic location (Fisher’s exact *P* = 0.51), and years since residency training (Fisher’s exact *P* = 0.72). There was a statistical significance between the preferred fractionation schedule and the number of SCLC patients treated in the department (Fisher’s exact *P* = 0.02) and by their own (Fisher’s exact *P* = 0.03). Moreover, the preferred fractionation schedule was highly correlated with the actual fractionation schedule of TRT (Kendall’s rank correlation tau *P* < 0.001), meaning that actual practice frequently aligned with preference.

We evaluated the treatment preferences of radiation oncologists, medical oncologists, and oncological surgeons to determine the variations in treatment preferences among specialties. As there was only one surgical oncologist among the respondents, only radiation oncologists and medical oncologists were analyzed. Among 174 radiation oncologists, 66.1% recommended QD TRT, 32.8% preferred BID TRT, and 1.1% advocated HFRT. For medical oncologists, 87.1% (27/31) recommended QD TRT, 1% supported BID TRT, and only one participant recommended HFRT, while 74.7% of radiation oncologists and 83.9% of medical oncologists utilize QD TRT more frequently in practice, with 23.6% vs. 9.7% who use BID TRT more frequently. There were no statistically significant correlations between the preferred fractionation schedule and demographic characteristics of respondents in either the radiation oncologist or medical oncologist groups, including practice setting (rad onc: Fisher’s exact P = 0.14; med onc: Fisher’s exact P = 0.25), geographic location (rad onc: Fisher’s exact P = 0.16; med onc: Fisher’s exact P = 0.654), years since residency training (rad onc: Fisher’s exact P = 0.30; med onc: Fisher’s exact P = 0.49), the number of SCLC patients treated in the department (rad onc: Fisher’s exact P = 0.65; med onc: Fisher’s exact P = 0.926), and by their own (Fisher’s exact P=1.00).

### Physician Reasoning for Fractionation Choice

**Figure 2:**
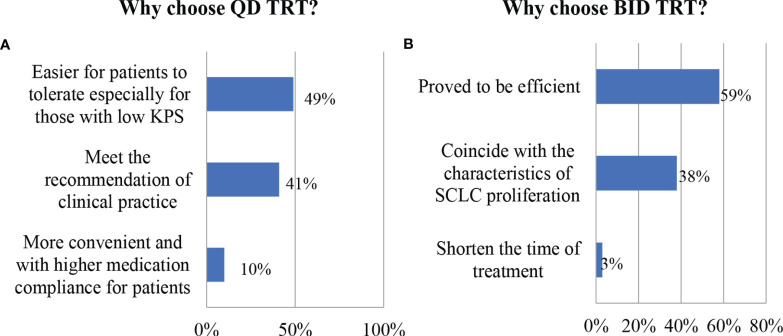
Physician reasoning for fractionation choice. **(A)** Reasons for those who choose QD TRT. **(B)** Reasons for those who choose BID TRT.

The results are summarized in **Figures 2A, B** for the reason that the respondents preferred different fractionation techniques. For those who preferred QD TRT, 15 out of 143 (10%) believed that QD TRT was more convenient and had a higher medication compliance for patients, and 58 of them (40.5%) reported that QD TRT met the recommendation of clinical practice, while nearly a half of them (48.9%) answered it was easier for patients to tolerate especially for those with low Karnofsky Performance Status (KPS). Nearly half of radiation oncologists (53.4%) agreed that QD TRT was easier for patients to tolerate, especially for those with low Karnofsky Performance Status (KPS), with 31.6% admitting that QD TRT met the clinical practice recommendations and 14.9% reporting that QD TRT was more convenient and had a higher medication compliance for patients. There was a 50/50 division among medical oncologists who believed that QD TRT was more tolerable for patients, particularly those with a pretty low general status and those who thought it was more consistent with clinical practice arrangements.

For those who preferred BID TRT, 23 out of 60 (38%) reported that BID TRT coincided with the characteristics of SCLC proliferation, 35 (59%) chose BID therapy for the reason that BID TRT has been proved to be efficient by INT 0096 and CONVERT trial, and only 2 respondents (3%) noted that BID TRT may shorten the time of treatment for patients who can tolerate radiotherapy. Regarding the reasons for choosing the BID TRT, 44.8% of radiation oncologists and 22.6% of medical oncologists believed that it had proven efficacy, 39.7% of radiation oncologists and 64.5% of medical oncologists believed that it was more compatible with the proliferative properties of SCLC, and 15.5% of radiation oncologists believed that it was more effective for tolerating shorter treatment cycles for patients.

Two of those who preferred HFRT said that HFRT met the recommendation of clinical practice, only 1 of them chose “other.”

### TRT Dose

**Figure 3:**
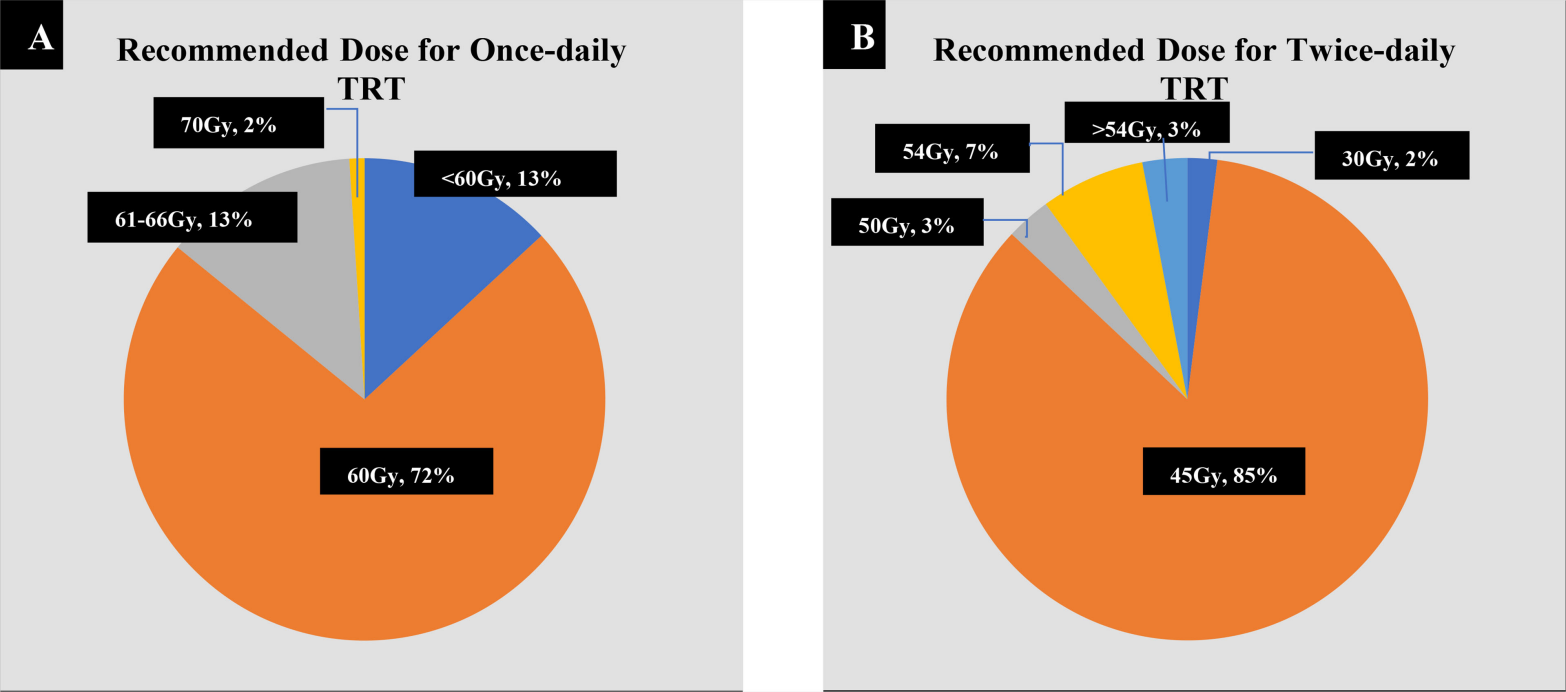
Preferred doses for once-daily (QD) and twice-daily (BID) thoracic radiotherapy (TRT). **(A)** For QD TRT, 103 out of 143 respondents (72%) recommended 60 Gy, 19 (13.2%) chose 61–66 Gy, 19 (13.2%) chose <60 Gy, and 2 (1.4%) chose 70 Gy. **(B)** For BID TRT, 51 out of 60 respondents (85.0%) recommended 45 Gy, 4 (6.7%) chose 54 Gy, 2 (3.3%) chose >54 Gy, and 1 (1.6%) chose 30 Gy.

When administering QD TRT, more than two-thirds (72%) of the respondents preferred a total dose of 60 Gy, followed by 13% opting for 61–66 Gy, 13% for <60 Gy, and 2% for 70 Gy (**Figure 3A**). When administering BID TRT, the majority (85%) of the respondents preferred a total dose of 45 Gy, with 2% choosing 30 Gy, 3% choosing 50 Gy, 7% choosing 54 Gy, and 3% choosing >54 Gy (**Figure 3B**). The majority of radiation oncologists (about 74.1%) agree that the total dose of QD TRT should be 60 Gy. Radiation oncologists proposing less than 60 and 61–66 Gy accounted for 9.8% and 14.9%, respectively, with only two out of 174 participants suggesting a total dose of 70 Gy. In the case of medical oncologists, 66.7% picked 60 Gy, whereas 19.4% and 16.1% chose a total dose of less than 60 and 61–66 Gy, respectively. For BID TRT dose selection, 83.9% of radiation oncologists and 54.8% of medical oncologists recommended a total dose of 45 Gy, and 8.0% vs. 9.7% chose 50 Gy, 5.2% vs. 16.1% chose 54 Gy, 1.1% vs. 12.9% recommended 30 Gy, and 1.7% vs. 6.5% advised a cumulative dose of more than 54 Gy.

For BID TRT, demographic characteristics including practice setting (Fisher’s exact *P* = 0.22), geographic location (Fisher’s exact *P* = 0.71), the number of SCLC patients treated in the department (Fisher’s exact *P* = 0.87), and the number of SCLC patients treated by their own (Fisher’s exact *P* = 0.79) were not correlated with the choice of dose, while the preferred TRT dose was highly correlated with the years since residency training (Fisher’s exact *P* = 0.009). Those experienced oncologists with over 10 years of experience after residency training were more likely to recommend doses of 45 Gy. For QD TRT, physicians with >10 years of experience after residency training and those who work in higher-level hospitals were more likely to recommend doses of 60 Gy (*P* < 0.001, *P* < 0.001). There were no statistically significant correlations between preferred TRT dose and demographic characteristics of respondents in either the radiation oncologist or medical oncologist groups, and P values were all higher than 0.05.

### Prophylactic Cranial Irradiation

When we asked the participants if PCI is necessary for LS-SCLC, 182 participants (90.3%) recommended PCI, 8 (3.8%) recommended that PCI is not necessary, while 16 (7.8%) chose “not sure.” Almost all of the respondents (99%) required patients to have a routine head MRI before PCI, and only 54 (26%) recommended administrating memantine. For those who recommended PCI, 142 (79%) chose 25 Gy as the total dose, 37 (20%) recommended 30 Gy, and only 2 (1%) chose the “other” option.

As for the timing of PCI, nearly 82% of participants thought that it should be performed when treatment is completed, 13% believed that PCI should begin immediately after concurrent chemo-radiotherapy, and 5% chose the “other” option.

Respondents were more likely (78%) to recommend PCI in patients who achieve complete clinical response (CR) after chemotherapy, 20% recommended partial response, and only 2% recommended stable disease.

### Angiogenesis Inhibitors

In our study, 52 out of 206 (26%) participants recommended concurrent anti-angiogenic therapy, while 154 (74%) did not. For those who recommended angiogenesis inhibitors, 49% preferred small-molecule TKI as the main treatment, followed by VEGFR antibodies (35%) and ENDOSTAR (16%). Those who work in higher-level hospitals and have over 10 years of experience after residency training were more likely not to recommend concurrent anti-angiogenic therapy (*P* = 0.037, *P* = 0.005).

### Immunotherapy

**Figure 4:**
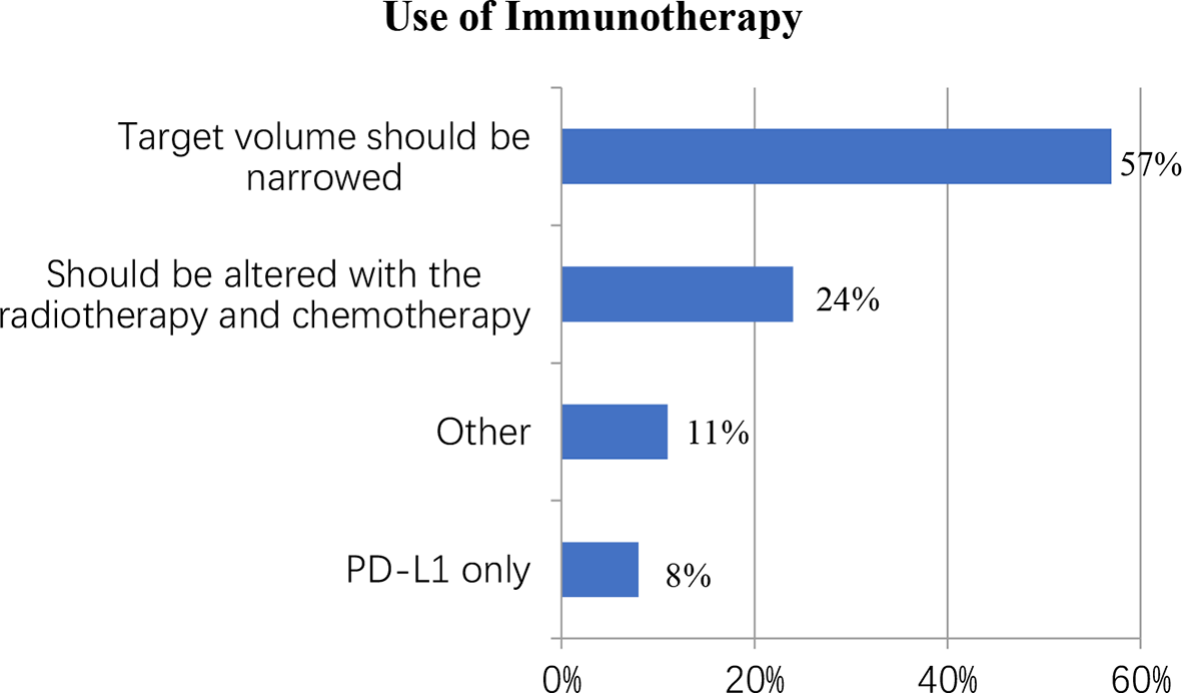
Physician reasoning for immunotherapy.

Despite the lack of unambiguous evidence supporting the use of immunotherapy in LS-SCLC, this study surveyed immunotherapy doctors to learn about the treatment in practice. Nearly half of the respondents (44%) said they were “unsure,” 32 (16%) said they were “not recommended,” and 84 (41%) said they would suggest immunotherapy with concurrent chemo-radiotherapy. When it came to those who preferred concurrent immunotherapy, 48 out of 84 (57%) said the target volume should be narrowed, 20 (24%) said the immunotherapy program should be altered with the radiotherapy and chemotherapy, 7 (8%) said PD-L1 only, and 9 (11%) chose “other” (**Figure 4**).

### Individualized Questions

Several questions about self-related knowledge were also included in the survey. Nearly 81% of participants were familiar with the guidelines or key clinical trials of SCLC. Almost all participants (96%) were enthusiastic about new developments in SCLC treatments. The last question is the proportion of patients with LS-SCLC treated according to the guidelines (including NCCN, CSCO, Chinese Medical Association) in China, and the results are summarized in **Table 2**.

**Table 2:** The proportion of patients with LS-SCLC treated according to the guidelines (including NCCN, CSCO, Chinese Medical Association).

The proportion of patients treated according to the guidelines	Respondents, n (%)
**<25%**	53 (26%)
**25%–50%**	69 (33%)
**50%–75%**	47 (23%)
**>75%**	38 (18%)

## Discussion

### Timing of TRT With Chemotherapy Cycles

In this study, 90% of participants recommended concurrent chemo-radiotherapy, and 92.2% chose to proceed TRT in cycle 1 or 2, which is consistent with NCCN guidelines, which require TRT to be administered concurrently with chemotherapy in cycle 1 or 2 (6). This result is in line with the findings of a study of 309 radiation oncologists in the United States, which found that 96% administered TRT with concurrent chemotherapy in cycles 1 and 2 and that over 98% of the 309 respondents recommended concurrent therapy over sequential therapy early in the regimen (12). In this study, those who recommended concurrent chemo-radiotherapy claimed that it can improve overall survival (OS) when compared to sequential chemo-radiotherapy. Even though some studies have shown that early TRT has specific survival advantages (13, 14), some studies have shown that “earlier or shorter” versus “later or longer” TRT had no effect on OS and that only early TRT combined with planned chemotherapy significantly improves 5-year OS (15, 16). While early concurrent chemo-radiotherapy improves OS, it comes at a substantial cost in terms of acute toxicity and particularly esophagitis (15). This case reveals the need for further investigation to improve survival and reduce toxicity in the treatment of LS-SCLC.

### Fractionation Schedule for LS-SCLC

In our study, 69% of respondents preferred TRT QD, 29% preferred BID fractionation, and 2% preferred HFRT. In actual practice, 76% chose QD TRT, 22 chose BID TRT, and 2% recommended HFRT, indicating that they were more inclined to choose QD TRT regardless of expert recommendations or in the actual practice process. This conclusion is consistent with the findings of a survey of US radiation oncologists on practice patterns of radiation dose and fractionation for LS-SCLC (7).

Although the NCCN guidelines recommend TRT for 45-Gy QD or 60–70-Gy BID for LS-SCLC patients (6), it is necessary to take “more convenient operation” and “better patient compliance” into account in actual practice. These realistic factors influence TRT fractionation schedule choices in both China and the United States (7). Although more oncologists are using QD TRT in their daily practice, the CONVERT trial argues that BID TRT should be considered the standard of care (5).

In the INT 0096 research, it is also considered that patients taking BID TRT have a considerably increased OS and 5-year survival rate (4). However, because the bioequivalent dosage of twice-daily chest radiation is larger than that of once-daily chest radiotherapy, the conclusion that BID TRT is superior to QD TRT remains disputed. Even though BID TRT was more effective than QD TRT in the INT 0096 research, there was a 36% local failure rate and a greater incidence of grade 3/4 esophagitis still exists (17).

There were also studies comparing hypofractionated radiation therapy (HFRT) and conventional segmentation (CFRT), with HFRT (40 Gy/15 fractions, 45 Gy/15 fractions, 45 Gy/20 fractions) and CFRT (60 Gy/30 or 66 Gy/33 fractions). The findings reveal that there is no difference in survival or toxicity between HFRT and CFRT, implying that HFRT might be used in place of CFRT (17). In the 2020 ASTRO annual meeting, a multicenter retrospective analysis compared three strategies of treatments—hypofractionated TRT (40 Gy/15f, QD TRT), conventional TRT (50 Gy/25f, QD TRT), and hyperfractionated TRT (45 Gy/30f, BID TRT)—but showed no differences of OS between three regimens (8). However, although the study monitored the patients from 2000 to 2016, only hypofractionated TRT was regularly used throughout the research period, whereas conventional TRT was largely utilized before 2006 and no longer used after 2009, with hyperfractionated not used until 2013. As a result, it may have an impact on the experiment’s outcome, and a longer timescale may be necessary to derive more reliable conclusions. In our study, only 2% of oncologists identified HFRT as a common choice in their daily work, indicating that further research is needed to determine whether HFRT has a higher benefit and whether it can replace CFRT. Moreover, a meta-analysis reported in the 2020 ASTRO annual meeting showed that SABR may be an eligible choice for inoperable early-stage, node-negative SCLC, while prospective studies are still needed for further evaluation (11).

In this study, for high-level hospitals, such as grade III level A hospitals (according to Chinese hospital classification standards), oncologists who treat more LS-SCLC patients in their daily work are more likely to prefer QD TRT over BID and HFRT. This not only implies that QD TRT is a more popular fractionation strategy in China, but it also indicates that, as the number of patients requiring TRT grows, QD TRT is a way to maximize medical resources. This study investigated the selection among different specialties. Although the proportion of radiation oncologists who picked QD TRT was only 66.7%, with that of medical oncologists being 87%, the differences may relate to the small number of medical oncologists (only 31). Nonetheless, there was no statistical association between the choice of different fractionation schedules and the participants’ fundamental information (geographic setting and location, etc.), and more data are still needed to draw accurate conclusions.

### TRT Dose

The NCCN recommends 2.0-Gy fractions over 6 to 7 weeks for a total dose of 60–70 Gy for QD TRT and 1.5-Gy fractions over 3 weeks for a total dose of 45 Gy for BID (6). Recently, the CALGB 30610 (Alliance)/RTOG 0538 trial demonstrated that although the high-dose QD TRT of 70 Gy did not significantly improve OS compared with BID TRT of 45 Gy, high dose once-daily TRT is still considered to be an acceptable option in LS-SCLC (10). In American analysis, the respondents preferred BID TRT and recommended a total dose of 45 Gy while the preferred dose of QD TRT was varied from 60 to 70 Gy, and only 10.0% of respondents recommended 70 Gy with most oncologists preferring lower than 70 Gy (7). In this study, only 2% of oncologists recommended 70 Gy, and those with more than 10 years of experience after residency training or who work in higher-level hospitals were more likely to recommend doses of 60 Gy, indicating that Chinese oncologists may be more conservative in their dose selection and that ongoing education of physicians throughout their careers is important. For BID TRT, the researchers in the American study believed that 45 Gy was overtreatment and there were no clinical trial results that supported higher doses at that time (7). In contrast, Bjorn Henning Gronberg et al. have compared the efficacy of standard-dose versus high-dose twice-daily thoracic radiotherapy (TRT) in small-cell lung cancer with limited disease (LD-SCLC) recently and have discovered that a higher radiotherapy dose of 60 Gy resulted in a significant survival improvement compared to 45 Gy without increased toxicity, implying that twice-daily thoracic radiotherapy of 60 Gy is an alternative schedule (9). In our study, more than two-thirds of respondents preferred a total dose of 60 Gy when administered QD TRT, whereas the majority of respondents preferred a total dose of 45 Gy when administered BID TRT, indicating that the choice of TRT dose is more affected by NCCN guidelines in China.

### Prophylactic Cranial Irradiation

In this study, almost all responding oncologists advised PCI and pre-PCI brain MRI for LS-SCLC patients for whom the disease was responding to initial therapy which met the recommendation of NCCN guidelines in which PCI should be performed especially for those who show complete or partial response to initial therapy (5). A randomized trial by the European Organisation for Research and Treatment of Cancer (EORTC) showed that PCI could decrease the rate of symptomatic brain metastases by 25.8% in patients with extensive-stage small-cell lung cancer (ES-SCLC) (18). However, the survival benefit of PCI disappeared when mandating brain MRI before randomization and at regular intervals, while for LS-SCLC, a recent analysis by Ozawa et al. (2015) showed that there were no significant survival benefits to PCI when MRI was used regularly pre-randomization (19). However, the trial mentioned above was a retrospective analysis and with a small size of sample size, indicating that further study is needed to approve the benefits of MRI and current patterns of MRI age.

### Use of Angiogenesis Inhibitors and Immunotherapy

Angiogenesis inhibition has been demonstrated to be an effective strategy in the treatment of a variety of tumors (20). Compared with non-small-cell lung cancer, SCLC has a higher microvessel count and VEGF overexpression, so anti-angiogenesis could represent a promising strategy of treatment for SCLC (21). Moreover, most of the clinical trials exploring the survival benefit of chemotherapy combined with antiangiogenic therapy are still conducted in patients with extensive-stage small-cell lung cancer (ES-SCLC). In 2007, the LUN90 trial evaluated the survival benefit of the combination of bevacizumab, irinotecan, and carboplatin in 51 patients with ES-SCLC but showed limit activity as first-line therapy (22). In the ECOG E3501 study, bevacizumab plus cisplatin and etoposide in patients with ES-SCLC could improve PFS and OS and have minimal increase in toxicities (23), while GOIRC-AIFA and SAUTE studies confirmed that bevacizumab improved PFS, but not OS (24, 25). For other angiogenesis inhibitors such as Endostar, sunitinib was not doing well in combination with chemotherapy in the first-line setting as well as ziv-aflibercept in combination with topotecan in the second-line setting (20). Only anlotinib was proved to improve OS and PFS as third-line therapy for Chinese ES-SCLC patients and approved indication in China (20), while treatment with angiogenesis inhibitors showed contrasting results in LS-SCLC patients. A phase II study analyzed bevacizumab as maintenance therapy following treatment with carboplatin, irinotecan, and concurrent radiation but failed to observe any positive results and concluded that bevacizumab was not suitable for maintenance therapy in patients with LS-SCLC. Moreover, there was a meta-analysis including 1,322 patients which showed that therapy with angiogenesis inhibitors did not enhance PFS, OS, or ORR but did increase the incidence of constipation and embolism in SCLC patients (26).

In our study, two-thirds of respondents did not recommend concurrent anti-angiogenetic therapy for LS-SCLC patients. For those who recommended, small-molecule TKI was the most popular choice. Moreover, it may be due to the approval of anlotinib as third-line treatment for ES-SCLC in China. Further study is still needed to determine the survival advantage of angiogenesis inhibitors combined with concurrent chemotherapy or radiotherapy in SCLC patients.

There was no evidence to suggest that radiotherapy with concurrent immunotherapy has benefits for SCLC patients. Randomized phase III trials, such as IMpower133 and CASPIAN, found that atezolizumab or durvalumab in combination with platinum-etoposide treatment enhanced overall survival in patients with advanced disease (27, 28), while the KEYNOTE-604 research found that pembrolizumab combined with chemotherapy did not improve overall survival (29). The use of radiation combined with immunotherapy in the treatment of SCLC remains “an area of active investigation” such as in NRG LU005 (https://clinicaltrials.gov/ct2/show/NCT03811002).

### Similarities and Differences Between US and Chinese Data

A large sample of radiation oncologists in the United States was investigated to better understand their decisions on the timing of radiation treatment beginning and fractionation schedule of LS-SCLC patients. Both US and Chinese data show that more oncologists actually recommend early concurrent radiotherapy with cycle 1 or 2 for patients with LS-SCLC, possibly due to NCCN guideline recommendation or data from clinical trials (e.g., phase III trials in Korea), meta-analyses, or systematic reviews supporting that early TRT can significantly improve patient survival (30). According to the US data, 60% of participants suggested QD TRT for LS-SCLC patients, and more than three-quarters of patients actually got QD TRT. Similarly, our study found that 69% of participants advocated QD TRT and that 76% of patients actually got QD TRT (7). QD TRT displayed remarkable simplicity of use for both patients and clinical application in both the US and China. Radiotherapy is frequently provided to Chinese patients in high-level hospitals, whereas radiotherapy facilities at county- or lower-level hospitals are still being established and upgraded to meet patient treatment. As a result, QD is a very frequent and appropriate choice for high-level institutions in order to satisfy as many patient treatment demands as possible while also increasing the efficiency of radiation equipment use. Nonetheless, BID TRT has not been entirely phased out in China. Moreover, these are facts that radiologists all around the world, not only in China and the United States, may have to face. There is still some variance in the dose, with both Chinese and US survey findings indicating that for BID TRT, a total dose of 45 Gy is the most recommended choice by experts, while for QD TRT, the dose is generally between 60 and 70 Gy. Interestingly, in the 2018 US trial, the treatment paradigm of increasing the total dose of QD TRT was proposed. However, the recent CALGB 30610 (Alliance)/RTOG 0538 study found that 70 Gy did not substantially enhance OS when compared to the 45-Gy BID regimen (10). As a result, the ideal dose of QD TRT may still be investigated further in the future.

### Limitations

This study has some limitations as well, such as a low response rate of just 216 valid replies. Furthermore, because the participants’ replies to the questions may be subjective, they may be vulnerable to some mistake owing to participant bias. As a result, our findings should be interpreted with caution. Although this study investigated and examined the choice of oncologists in various specialties, it was not able to assess the choice of the surgeon since only one answer was obtained. Furthermore, due to the gap in the number of medical oncologists vs. radiation oncologists, follow-up studies are still required to extend the data and reach more accurate findings. Our team will continue to update the data in order to obtain more accurate and thorough results.

## Conclusion

The goal of this survey was to broadly sample oncologists in China on their management of LS-SCLC. Despite differences in LS-SCLC treatment plans, our survey found that most Chinese oncologists still follow the NCCN guidelines in practice, namely, early timing of (QD) TRT in 2.0-Gy fractions over 6 to 7 weeks for a total dose of 60 Gy with a universal endorsement of PCI and pre-PCI brain MRI practices recommended. This study created a practice pattern baseline for future clinical trials for patients with LS-SCLC, provided Chinese oncologists with an agreement on management, and was of reference significance for radiation oncologists worldwide in the treatment of small-cell lung cancer.

## Data Availability Statement

The raw data supporting the conclusions of this article will be made available by the authors, without undue reservation.

## Author Contributions

CX: conceptualization, formal analysis, writing—original draft. ML: methodology, writing—original draft. XC, SY, and JC: data collection and curation, project administration. SZ, MC, NB, and XH: data collection and curation. JL, WZ, and PW: data collection. LZ and NL: supervision, validation, writing—review and editing. All authors contributed to the article and approved the submitted version.

## Conflict of Interest

The authors declare that the research was conducted in the absence of any commercial or financial relationships that could be construed as a potential conflict of interest.

## Publisher’s Note

All claims expressed in this article are solely those of the authors and do not necessarily represent those of their affiliated organizations, or those of the publisher, the editors and the reviewers. Any product that may be evaluated in this article, or claim that may be made by its manufacturer, is not guaranteed or endorsed by the publisher.

## References

[1] Van Meerbeeck JP Fennell DA De Ruysscher DKM. Small-Cell Lung Cancer. Lancet (2011) 378:1741–55. doi: 10.1016/S0140-6736(11)60165-7 21565397

[2] Wang P Zou J Wu J Zhang C Ma C Yu J. Clinical Profiles and Trend Analysis of Newly Diagnosed Lung Cancer in a Tertiary Care Hospital of East China During 2011-2015. J Thorac Dis (2017) 9(7):1973–9. doi: 10.21037/jtd.2017.06.102 PMC5542980 28839996

[3] Sabari JK Lok BH Laird JH Poirier JT Rudin CM. Unravelling the Biology of SCLC: Implications for Therapy. Nat Rev Clin Oncol (2017) 14:549–61. doi: 10.1038/nrclinonc.2017.71 PMC5843484 28534531

[4] Proudhom MA Noël G Mazeron JJ. Twice-Daily Compared With Once-Daily Thoracic Radiotherapy in Limited Small-Cell Lung Cancer Treated Concurrently With Cisplatin and Etoposide. Phase III Comparison of Twice-Daily Split Course Irradiation Versus Once-Daily Irradiation for Patients With Limited Stage Small-Cell Lung Carcinoma. Cancer Radiother (2000) 4:319–20. 10994398

[5] Faivre-Finn C Snee M Ashcroft L Appel W Barlesi F Bhatnagar A. Concurrent Once-Daily Versus Twice-Daily Chemoradiotherapy in Patients With Limited-Stage Small-Cell Lung Cancer (CONVERT): An Open-Label, Phase 3, Randomised, Superiority Trial. Lancet Oncol (2017) 18(8):1116–25. doi: 10.1016/S1470-2045(17)30318-2 PMC5555437 28642008

[6] Kalemkerian GP Loo BW Akerley W Attia A Bassetti M Boumber Y. NCCN Guidelines Insights: Small Cell Lung Cancer, Version 2.2018. J Natl Compr Canc Netw (2018) 16(10):1171–82. doi: 10.6004/jnccn.2018.0079 30323087

[7] Farrell MJ Yahya JB Degnin C Chen Y Holland JM Henderson MA. Radiation Dose and Fractionation for Limited-Stage Small-Cell Lung Cancer: Survey of US Radiation Oncologists on Practice Patterns. Clin Lung Cancer (2019) 20(1):13–9. doi: 10.1016/j.cllc.2018.08.015 30219240

[8] Almahmudi M Atwal P Casey S Canlas R Hsu F. Pattern of Practice and Comparison of Thoracic Radiotherapy for the Radical Treatment of Limited-Stage Small Cell Lung Cancer. Int J Radiat OncologyBiologyPhysics (2020) 108:e117–8. doi: 10.1016/j.ijrobp.2020.07.1248

[9] Grønberg BH Killingberg KT Fløtten Ø Brustugun OT Hornslien K Madebo T. High-Dose Versus Standard-Dose Twice-Daily Thoracic Radiotherapy for Patients With Limited Stage Small-Cell Lung Cancer: An Open-Label, Randomised, Phase 2 Trial. Lancet Oncol (2021) 22(3):321–31. doi: 10.1016/S1470-2045(20)30742-7 33662285

[10] Bogart JA Wang XF Masters GA Gao J Komaki R Charles S. Phase 3 Comparison of High-Dose Once-Daily (QD) Thoracic Radiotherapy (TRT) With Standard Twice-Daily (BID) TRT in Limited Stage Small Cell Lung Cancer (LSCLC): CALGB 30610 (Alliance)/RTOG 0538. (2021) 39:8505–5. doi: 10.1200/JCO.2021.39.15_suppl.8505

[11] Safavi AH Mak D Boldt G Chen H Louie AV. Stereotactic Ablative Radiotherapy in T1-2n0m0 Small Cell Lung Cancer: A Systematic Review and Meta-Analysis. Int J Radiat OncologyBiologyPhysics (2020) 108:e106–7. doi: 10.1016/j.ijrobp.2020.07.1225

[12] Farrell MJ Yahya JB Degnin C Chen Y Holland JM Henderson MA. Timing of Thoracic Radiation Therapy With Chemotherapy in Limited-Stage Small-Cell Lung Cancer: Survey of US Radiation Oncologists on Current Practice Patterns. Clin Lung Cancer (2018) 19(6):e815–21. doi: 10.1016/j.cllc.2018.04.007 29857969

[13] Hu X Xia B Bao Y Xu YJ Wang J Ma HL. Timing of Thoracic Radiotherapy is More Important Than Dose Intensification in Patients With Limited-Stage Small Cell Lung Cancer: A Parallel Comparison of Two Prospective Studies. Strahlenther Onkol (2020) 196(2):172–81. doi: 10.1007/s00066-019-01539-1 31784801

[14] Takada M Fukuoka M Kawahara M Sugiura T Yokoyama A Yokota S. Phase III Study of Concurrent Versus Sequential Thoracic Radiotherapy in Combination With Cisplatin and Etoposide for Limited-Stage Small-Cell Lung Cancer: Results of the Japan Clinical Oncology Group Study 9104. J Clin Oncol (2002) 20(14):3054–60. doi: 10.1200/JCO.2002.12.071 12118018

[15] De Ruysscher D Lueza B Le Péchoux C Johnson DH O'Brien M Murray N. Impact of Thoracic Radiotherapy Timing in Limited-Stage Small-Cell Lung Cancer: Usefulness of the Individual Patient Data Meta-Analysis. Ann Oncol (2016) 27(10):1818–28. doi: 10.1093/annonc/mdw263 PMC5035783 27436850

[16] Kubota K Hida T Ishikura S Mizusawa J Nishio M Kawahara M. Etoposide and Cisplatin Versus Irinotecan and Cisplatin in Patients With Limited-Stage Small-Cell Lung Cancer Treated With Etoposide and Cisplatin Plus Concurrent Accelerated Hyperfractionated Thoracic Radiotherapy (JCOG0202): A Randomised Phase 3 Study. Lancet Oncol (2014) 15(1):106–13. doi: 10.1016/S1470-2045(13)70511-4 24309370

[17] Zayed S Chen H Ali E Rodrigues GB Warner A Palma DA. Is There a Role for Hypofractionated Thoracic Radiation Therapy in Limited-Stage Small Cell Lung Cancer? A Propensity Score Matched Analysis. Int J Radiat Oncol Biol Phys (2020) 108(3):575–86. doi: 10.1016/j.ijrobp.2020.06.008 PMC7293491 32544575

[18] Takahashi T Yamanaka T Seto T Harada H Nokihara H Saka H. Prophylactic Cranial Irradiation Versus Observation in Patients With Extensive-Disease Small-Cell Lung Cancer: A Multicentre, Randomised, Open-Label, Phase 3 Trial. Lancet Oncol (2017) 18(5):663–71. doi: 10.1016/S1470-2045(17)30230-9 28343976

[19] Ozawa Y Omae M Fujii M Matsui T Kato M Sagisaka S. Management of Brain Metastasis With Magnetic Resonance Imaging and Stereotactic Irradiation Attenuated Benefits of Prophylactic Cranial Irradiation in Patients With Limited-Stage Small Cell Lung Cancer. BMC Cancer (2015) 15:589. doi: 10.1186/s12885-015-1593-2 26275617 PMC4537586

[20] Montanino A Manzo A Carillio G Palumbo G Esposito G Sforza V. Angiogenesis Inhibitors in Small Cell Lung Cancer. Front Oncol (2021) 11:655316. doi: 10.3389/fonc.2021.655316 34123809 PMC8195287

[21] Lucchi M Mussi A Fontanini G Faviana P Ribechini A Angeletti CA. Small Cell Lung Carcinoma (SCLC): The Angiogenic Phenomenon. Eur J Cardiothorac Surg (2002) 21:1105–10. doi: 10.1016/S1010-7940(02)00112-4 12048093

[22] Spigel DR Hainsworth JD Simons L Meng C Burris HA Yardley DA. Irinotecan, Carboplatin, and Imatinib in Untreated Extensive-Stage Small-Cell Lung Cancer: A Phase II Trial of the Minnie Pearl Cancer Research Network. J Thorac Oncol (2007) 2(9):854–61. doi: 10.1097/JTO.0b013e31814617b7 17805064

[23] Horn L Dahlberg SE Sandler AB Dowlati A Moore DF Murren JR Schiller JH. Phase II Study of Cisplatin Plus Etoposide and Bevacizumab for Previously Untreated, Extensive-Stage Small-Cell Lung Cancer: Eastern Cooperative Oncology Group Study E3501. J Clin Oncol (2009) 27(35):6006–11. doi: 10.1200/JCO.2009.23.7545 PMC2793043 19826110

[24] Baldini E Cinieri S Brighenti M Zanelli F Defraia E Chiari R. Italian, Multicenter, Phase III, Randomized Study of Cisplatin Plus Etoposide With or Without Bevacizumab as First-Line Treatment in Extensive-Disease Small-Cell Lung Cancer: The GOIRC-AIFA FARM6PMFJM Trial. J Clin Oncol (2017) 35(12):1281–7. doi: 10.1200/JCO.2016.69.4844 28135143

[25] Spigel DR Townley PM Waterhouse DM Fang L Adiguzel I Huang JE. Randomized Phase II Study of Bevacizumab in Combination With Chemotherapy in Previously Untreated Extensive-Stage Small-Cell Lung Cancer: Results From the SALUTE Trial. J Clin Oncol (2011) 29(16):2215–22. doi: 10.1200/JCO.2010.29.3423 21502556

[26] Li Q Wu T Jing L Li MJ Tian T Ruan ZP. Angiogenesis Inhibitors for the Treatment of Small Cell Lung Cancer (SCLC): A Meta-Analysis of 7 Randomized Controlled Trials. Med (Baltimore) (2017) 96(13):e6412. doi: 10.1097/MD.0000000000006412 PMC5380252 28353568

[27] Paz-Ares L Dvorkin M Chen Y Reinmuth N Hotta K Trukhin D. Durvalumab Plus Platinum-Etoposide Versus Platinum-Etoposide in First-Line Treatment of Extensive-Stage Small-Cell Lung Cancer (CASPIAN): A Randomised, Controlled, Open-Label, Phase 3 Trial. Lancet (2019) 394(10212):1929–39. doi: 10.1016/S0140-6736(19)32222-6 31590988

[28] Liu SV Reck M Mansfield AS Mok T Scherpereel A Reinmuth N. Updated Overall Survival and PD-L1 Subgroup Analysis of Patients With Extensive-Stage Small-Cell Lung Cancer Treated With Atezolizumab, Carboplatin, and Etoposide (Impower133). J Clin Oncol (2021) 39(6):619–30. doi: 10.1200/JCO.20.01055 PMC8078320 33439693

[29] Rudin CM Awad MM Navarro A Gottfried M Peters S Csõszi T. Pembrolizumab or Placebo Plus Etoposide and Platinum as First-Line Therapy for Extensive-Stage Small-Cell Lung Cancer: Randomized, Double-Blind, Phase III KEYNOTE-604 Study. J Clin Oncol (2020) 38(21):2369–79. doi: 10.1200/JCO.20.00793 PMC7474472 32468956

[30] Lu H Fang L Wang X Cai J Mao W. A Meta-Analysis of Randomized Controlled Trials Comparing Early and Late Concurrent Thoracic Radiotherapy With Etoposide and Cisplatin/Carboplatin Chemotherapy for Limited-Disease Small-Cell Lung Cancer. Mol Clin Oncol (2014) 2:805–10. doi: 10.3892/mco.2014.311 PMC4106745 25054049

