# Supplementary material for: Practice Patterns of Treatment Strategy of Limited-Stage Small-Cell Lung Cancer: Survey of Chinese Oncologists
Source: Front Oncol. 2022 May 12;12:872324. doi: 10.3389/fonc.2022.872324 (PMC9149654; doi:10.3389/fonc.2022.872324)
Supplement: Supplementary file 1 [file DataSheet_1.docx]

Q1: Your present professional directorate is?

1. Radiation oncology
2. Oncology surgery
3. Medical oncology
4. Other________

Q2: Classification of institutions

1. Tertiary hospital
2. Grade II hospital
3. Grade III level A hospital
4. Grade III level B hospital
5. Other________

Q3: Your professional title is?

1. Chief physician
2. Associate chief physicians
3. Attending physicians
4. Resident
5. Other________

Q4: Your practice region is?

1. North
2. Central
3. East
4. South
5. Northeast
6. Northwest
7. Southwest
8. Other________

Q5: Duration since residency training

1. <5 years
2. 5-10 years
3. >10 years

Q6: Number of LS-SCLC patients treated per year in the department

1. 10-40
2. 41-70
3. 71-100
4. >100

Q7: How many LS-SCLC patients do you treat per year?

1. 10-40
2. 41-70
3. 71-100
4. >100

Q8: Number of ES-SCLC patients treated per year in the department

1. 10-40
2. 41-70
3. 71-100
4. >100

Q9: How many ES-SCLC patients do you treat per year

1. 10-40
2. 41-70
3. 71-100
4. >100

Q10: Number of LSCLC patients on initial visit

1. 10-40
2. 41-70
3. 71-100
4. >100

Q11: Do you recommend thoracic radiotherapy in LS-SCLC patients?

1. Yes
2. No
3. Unknown

Q12: Do you recommend thoracic radiotherapy in ES-SCLC patients?

1. Yes
2. No
3. Unknown

Q13: If you recommend thoracic radiotherapy in LS-SCLC patients, when do you prefer to start thoracic radiotherapy?

1. After 1 circle of chemotherapy
2. After 2 circles of chemotherapy
3. After 3 circles of chemotherapy
4. Other________

Q14: When would you like to begin thoracic radiotherapy in practice for LS-SCLC?

1. After 1 circle of chemotherapy
2. After 2 circles of chemotherapy
3. After 3 circles of chemotherapy
4. Other________

Q15: If you recommend thoracic radiotherapy after 1 circle of chemotherapy, do you think that it is better than that after 2 or 3 circles?

1. Yes
2. No
3. Unknown

Q16: If you recommend thoracic radiotherapy after 1 circle of chemotherapy, how do you design the GTV?

1. Computer Tomography before chemotherapy
2. Computer Tomography after chemotherapy

Q17: If you recommend thoracic radiotherapy after 1 circle of chemotherapy, how do you design the CTV?

1. Computer Tomography before chemotherapy
2. Computer Tomography after chemotherapy

Q18: How do you determine CTV of thoracic radiotherapy of LS-SCLC?

1. Enlarged lymphatic drainage area
2. Affected lymphatic drainage area

Q19: Which do you prefer? Concurrent chemo-radiotherapy or sequential chemo-radiotherapy?

1. concurrent chemo-radiotherapy
2. sequential chemo-radiotherapy

Q20: Do you think concurrent chemo-radiotherapy has a survival advantage over sequential chemo-radiotherapy?

1. Yes
2. No
3. Unknown

Q21: Which schedule do you prefer: concurrent chemo-radiotherapy or sequential chemo-radiotherapy?

1. concurrent chemo-radiotherapy
2. sequential chemo-radiotherapy

Q22: Which fractionation schedule do you prefer: QD, BID or HFRT?

1. QD
2. BID
3. HFRT

Q23: The reason you chose QD thoracic radiotherapy.

2. meet the recommendation of clinical practice
3. easier for patients to tolerate, especially for those with low Karnofsky Performance Status
4. other________

Q24: The reason you chose BID thoracic radiotherapy.

1. coincided with the characteristics of SCLC proliferation
2. proved to be efficient by INT 0096 and CONVERT trial
3. shorten the time of treatment for patients who can tolerate radiotherapy

Q25: Your recommended dose for QD thoracic radiotherapy.

1. <60Gy
2. 60Gy
3. 60-66Gy
4. 70Gy
5. Other________

Q26: Your recommended dose for BID thoracic radiotherapy.

1. 30Gy
2. 45Gy
3. 50Gy
4. 54Gy
5. >54Gy
6. Other________

Q27: Do you recommend a gradient to separate GTV and CTV doses

1. Yes
2. No
3. Unknown

Q28: In the institute you are practicing, which fractionation schedule is often used?

1. QD
2. BID
3. HFRT
4. Other________

Q29: Do you think Prophylactic Cranial Irradiation (PCI) is necessary for LS-SCLC?

1. Yes
2. No
3. Unknown

Q30: Would you recommend PCI?

1. Yes
2. No
3. Unknown

Q31: What time is best for LS-SCLC patients to administrate PCI?

1. when treatment completed
2. should begin immediately after concurrent chemo-radiotherapy
3. other________

Q32: What kinds of patient would you recommend to administrate PCI?

1. CR
2. PR
3. Other________

Q33: Do you think it is necessary for ES-SCLC patients to administrate PCI?

1. Yes
2. No
3. Unknown

Q34: If you recommend PCI, when would you recommend to perform for ES-SCLC?

1. when treatment completed
2. should begin immediately after concurrent chemo-radiotherapy
3. other________

Q35: Do you think it is necessary to administrate MRI before PCI?

1. Yes
2. No
3. Unknown

Q36: Would you ask the patient for a follow-up MRI after PCI?

1. Yes
2. No
3. Unknown

Q37: Dose for PCI

1. 25Gy
2. 30Gy
3. Other_______

Q38: Would you recommend administrating Memantine after PCI?

1. Yes
2. No
3. Unknown

Q39: What do you think affects the prognosis of SCLC patients (multiple choice)

1. Tumor size
2. Lymph node metastasis or not
3. Start time of radiotherapy
4. Whether administrate concurrent chemo-radiotherapy or not
5. Whether to perform PCI
6. Start time of PCI
7. D-dimer level
8. Smoking
9. Other________

Q40: Are you familiar with important literature on SCLC?

1. Yes
2. No
3. Unknown

Q41: Are you keeping an eye on the latest advances in the treatment of SCLC?

1. Yes
2. No
3. Unknown

Q42: The percentage of SCLC patients you treated receiving immunotherapy was

1. <25%
2. 25-50%
3. 50-75%
4. >75%
5. Other________

Q43：Do you think the target volume of thoracic radiotherapy for extensive-stage SCLC in the era of immunotherapy needs to be changed?

1. Yes
2. No
3. Unknown

Q44：Which fractionation schedule do you prefer？

1. QD
2. BID
3. HFRT

Q45：Do you recommend antiangiogenic therapy concurrently in SCLC treatment?

1. Yes
2. No
3. Unknown

Q46：Which antiangiogenic therapy would you prefer?

1. Small molecule VEGFR inhibitors
2. Monoclonal antibody targeting VEGFR
3. Endostar
4. Other________

Q47：Would you think it is necessary for LS-SCLC patients to administrate concurrent immunotherapy?

1. Yes
2. No
3. Unknown

Q48: If concurrent chemoradiotherapy combined with immunotherapy has been treated in LS-SCLC patients, what do you think should be changed?

1. Not recommended
2. Chemotherapy drugs and regimens
3. Radiotherapy target volume should be reduced
4. Only PDL1 can be used
5. Other________

Q49：The proportion of LS-SCLC patients currently treated according to guidelines (including NCCN, CSCO, Chinese Medical Association) in China

1. <25%
2. 25-50%
3. 50-75%
4. >75%

Q50: The proportion of ES-SCLC patients currently treated according to guidelines (including NCCN, CSCO, Chinese Medical Association) in China

1. <25%
2. 25-50%
3. 50-75%
4. >75%
